# Nasopharyngeal and oral microbiota profiling in SARS-CoV-2 infected pregnant women

**DOI:** 10.1038/s41598-025-19344-5

**Published:** 2025-10-09

**Authors:** Niccolò Giovannini, Alessia Limena, Carolina Ercolino, Sara Colonia Uceda Renteria, Francesco Strati, Maria Rita Giuffrè, Paola Maragno, Ilma Floriana Carbone, Federica Facciotti, Ferruccio Ceriotti, Enrico Ferrazzi, Debora Lattuada

**Affiliations:** 1 Department “Area Materno-Infantile”, Fondazione IRCCS Ca’Granda Ospedale Maggiore Policlinico, Milan, Italy; 2 Centro di Ricerche Coordinate Medicina Materno Fetale e Neonatale, University of Milan, Milan, Italy; 3 Virology Unit, Foundation IRCCS Ca’ Granda Ospedale Maggiore Policlinico, Milan, Italy; 4 Department of Biotechnology and Biosciences, University of Milan Bicocca, Milan, Italy; 5 Department of Experimental Oncology, European Institute of Oncology IRCCS, Milan, Italy; 6 Gastroenterology and Endoscopy Unit, Ospedale Maggiore Policlinico, Fondazione IRCCS Cà Granda, Milan, Italy

**Keywords:** SARS-CoV-2, Pregnancy, Nasopharyngeal and oral microbiota, Infection, Bacteria, Clinical microbiology, Infectious-disease diagnostics, Viral infection

## Abstract

Variations have been found in the upper respiratory tract microbiota in SARS-CoV-2 positive patients compared to healthy subjects, with different dominant species and diversity indexes detected, including a decrease in biodiversity and an increased abundance of bacterial pathogens. Moreover, these discrepancies were observed in patients with both mild and severe symptoms. Notably, the inflammatory state appears to be significantly influenced by the characteristics of the indigenous microbiota. This is particularly interesting in pregnant patients, as pregnancy involves an adaptive adjustment of the microbiota due to hormonal changes aimed at providing immune protection. The relationship between the microbiota of pregnant women and SARS-CoV-2 has not been deeply explored so far. The purpose of the present study is to investigate the relationship between SARS-CoV-2, nasopharyngeal and oral microbiota, and pregnancy. To our knowledge this is the first simultaneous investigation on both nasopharyngeal and oral microbiota in SARS-Cov-2 infected pregnant women. In this study, the nasopharyngeal and oral microbiota were analysed in 43 women in their third trimester of pregnancy enrolled from April 2020 to February 2021. The differential abundance of taxa was tested and alpha and beta diversity were evaluated. SARS-CoV-2 infected pregnant women showed an alteration of the nasopharyngeal and oral microbiota compared to healthy pregnant women. In both the nasopharyngeal and oral microbiota of the SARS-CoV-2 infected pregnant women, we found a variation in taxa, represented by an enrichment of pathobionts, which increased particularly with the severity of symptoms. Specifically, a significant reduction in microbial biodiversity has been identified within the nasopharyngeal microbiota of SARS-CoV-2 positive women. Furthermore, enrichment in pathobionts was noted in both asymptomatic and symptomatic women, with these changes being more pronounced in the nasopharyngeal microbiota compared to the oral one. The nasopharyngeal microbiota of asymptomatic and symptomatic SARS-CoV-2 infected women showed an enrichment of pathogens and pathobionts such as Corynebacterium, Fusobacterium, Neisseria, Streptococcus, Haemophilus, Mycobacterium and Porphyromonas compared with the control group. The oral microbiota showed an enrichment of pathobionts such as Neisseria, Fusobacterium and Streptococcus. A random forest classifier applied to metagenomic data from nasopharyngeal and oral swabs showed that the nasopharyngeal microbiota is the best sampling site to predict the patients’ SARS-CoV-2 infection status. Gulbenkiania, Burkholderia and Actinomyces, all taxa significantly enriched in the control group compared to SARS-CoV-2 infected women, were the most important features selected by the classifier. Finally, correlations between the nasopharyngeal and oral microbiota and clinical parameters of pregnant women, particularly BMI and procalcitonin, were observed. SARS-CoV-2 infected pregnant women showed an alteration of the nasopharyngeal and oral microbiota compared to healthy pregnant women. We found a variation in taxa, represented by the enrichment of pathobionts in both the nasopharyngeal and oral microbiota of SARS-CoV-2 infected pregnant women, particularly increased in symptomatic individuals. The nasopharyngeal microbiota appears to be a better predictor of SARS-CoV-2 infection and its severity than the oral microbiota.

**Supplementary Information:**

The online version contains supplementary material available at 10.1038/s41598-025-19344-5.

## Introduction

SARS-CoV2 is an RNA Coronavirus responsible for COVID-19, associated with the recent syndemic. This emerging global health threat has provided an opportunity to further understand of the biological interactions between host microbiota and the environment. The upper respiratory tract (URT) is the primary entry point for the virus, and its resident microbiota can influence viral spread and disease progression.

Several studies have found differences in URT microbiota between SARS-CoV-2 infected patients and healthy individuals, revealing different dominant species and diversity indexes, with a decrease in biodiversity and an increased abundance of bacterial pathogens in infected patients^1–4^. Furthermore, patients with severe symptoms exhibited different URT microbiota compared to those with mild symptoms^1,5^. The inflammatory modulation by the microbiota as a host defense could explain the varying immune responses, ranging from over-reaction to under-reaction, especially in severe cases of SARS-CoV-2 disease. A systematic review published in 2020 on SARS-CoV-2 infection and pregnancy outcomes showed a slight increase in maternal and fetal risks^6^. Most first-wave infections occurred in the third trimester and were associated with a small increase in hospitalization, admission to the intensive care unit, mechanical ventilation, preterm birth, higher cesarean section rates, and low birth weight^6^.

These complications may be due to the remodelling of the maternal immune system during pregnancy. A pro-inflammatory state is observed in the 1st and 3rd trimesters, favouring implantation and the onset of labour, while an anti-inflammatory state in the 2nd trimester facilitates fetal growth^7,8^.

The inflammatory state appears to be significantly influenced by the characteristics of the microbiota. Specifically, during pregnancy, the microbiota undergoes adaptive changes due to hormonal shifts to provide immune protection^9,10^.

Despite extensive researches, the relationship between the upper respiratory tract microbiota of pregnant women and SARS-CoV-2 has not been deeply explored.

The purpose of the present study is to investigate nasopharyngeal and oral microbiota profiles in SARS-CoV-2 infected pregnant women, stratified by clinical severity, compared to controls, and to investigate which one of the two microbiota is the better predictor of SARS-CoV-2 infection and its severity.

## Methods

### Patient recruitment

This was a monocentric, longitudinal, prospective study conducted on 43 SARS-CoV-2 unvaccinated pregnant women between 31 and 40 weeks of gestation. Patients were enrolled from April 2020 to February 2021 at Foundation IRCCS Ca’ Granda Ospedale Maggiore Policlinico in Milan, Italy. None of the participants used any anti-infection drugs prior to sample collection. Since March 2020, all pregnant women admitted to the hospital were tested for SARS-CoV-2 infection using rRT-PCR on nasopharyngeal swabs, and those who agreed to participate in the study were assigned to either the SARS-CoV-2 infected or non-infected group based on their test results. In this study, the SARS-CoV-2 infected group was further divided into asymptomatic and symptomatic subgroups based on clinical complications. The study was approved by the Ethics Committee Milan Area 2 of Foundation IRCCS Ca’ Granda Ospedale Maggiore Policlinico (N.1651). Written informed consent was obtained from all participants.

### Sample collection and clinical data

After obtaining consent, nasopharyngeal and oral swabs were collected from each participant at the time of hospital admission for microbiota analysis. Nasopharyngeal and oropharyngeal swabs were collected using COPAN’s Flocked Swabs (FLOQSwabs Inside, Copan, Italy), and samples were placed in eSwab – Copan’s Liquid Amies Elution Swab (Copan, Italy), following the manufacturer instructions. Specifically, for the nasopharyngeal swab, the Flocked Swabs were inserted into both nostrils up to the posterior wall of the nasopharynx, gently rotated and withdrawn, and then placed in the eSwab. Oral swabs were obtained by scraping the lower and upper sub-gingival spaces with the Flocked Swabs and collected in the eSwab.

Nasopharyngeal and oral swabs were stored at − 80 °C until DNA extraction.

Women involved in the study completed a questionnaire to assess their eating habits, oral hygiene, lifestyle, medications, and any pregnancy or non-pregnancy related diseases.

Blood tests were performed for a complete blood count, procalcitonin (PCT), and PCR. Patients undergoing antibiotic therapy were excluded. Pregnant patients who tested positive were classified as asymptomatic if no symptoms were reported, or as symptomatic if they presented one or more of the following symptoms: gastrointestinal (diarrhea), mild respiratory (cough and pharyngodynia), severe respiratory impairment (dyspnea and chest pain), fever, ageusia, anosmia and arthralgia. Supplementary Table S1 shows sample clinical data of 43 pregnant women enrolled in this study.

#### Nucleic acid isolation

Total nucleic acid extraction was performed using the ReliaPrep™ gDNA Miniprep System (Promega, USA). Briefly, 1 ml of nasopharyngeal swab buffer was added to 500 ml of CTAB buffer and incubated at 95 °C for 5′. Then, 40 µl of Proteinase K was added and incubated at 70 °C for 10 min. After a 5-minute spin, the supernatant was added to the same volume of Lysis Buffer and then mixed with twice the volume of Isopropanol. The sample was loaded onto the ReliaPrep™ Binding Column following the manufacturer’s protocol. The elution volume was 50 µl. Extracts were stored at − 80 °C prior to 16 S rRNA sequencing.

#### Microbiota profiling

16 S rRNA gene amplification, library preparation, and paired-end sequencing on the Illumina MiSeq platform were performed as previously described^11,12^. The 16 S rRNA gene was amplified using region-specific primers targeting the V3 (5′- CCTACGGGNGGCWGCAG-3′) and V4 (5′- GACTACHVGGGTATCTAATCC-3′) regions of the 16 S rRNA gene. Primer sequences were selected from Klindworth et al.^13^.

Reads were pre-processed using the MICCA pipeline (v.1.7.0) (https://micca.readthedocs.io/en/latest/index.html)^14^. Forward and reverse primers trimming and quality filtering were performed using micca trim and micca filter, respectively. Filtered sequences were denoised using the UNOISE (RC, 2016) algorithm implemented in micca otu to determine true biological sequences at the single-nucleotide resolution by generating amplicon sequence variants (ASVs) (Table S2 and OTUs.fasta). Bacterial ASVs were taxonomically classified using micca classify and the Ribosomal Database Project (RDP) Classifier v2.13^15^ (Table S3). Multiple sequence alignment (MSA) of 16 S rRNA gene sequences was performed using the Nearest Alignment Space Termination (NAST) algorithm^16^ implemented in micca msa with the template alignment clustered at 97% similarity of the Greengenes database^17^ (release 13_08). Phylogenetic trees were inferred using micca tree^18^. Sampling heterogeneity was reduced by rarefying samples to the depth of the least abundant sample using micca tablerare. Alpha (within-sample richness) and beta-diversity (between-sample dissimilarity) estimates were computed using the phyloseq R package^19^. Alpha-diversity was evaluated using the Shannon index. The Wilcoxon rank-sum test was performed to test the significance of paired richness differences. Beta-diversity analysis was evaluated using the Unweighted UniFrac distance, the Weighted UniFrac distance, and the Bray-Curtis dissimilarity index. PERMANOVA (Permutational multivariate analysis of variance), a non-parametric multivariate statistical permutation test, was performed to test the significance between groups. The PERMANOVA test was performed using the adonis function in the R package vegan with 999 permutations. ASVs differential abundance testing was carried out using the R package DESeq2^20^ with non-rarefied data^21^. Spearman’s correlations were tested using the psych R package^22^. Random Forest analyses of 16 S rRNA gene sequencing data were performed using the randomForest R package^23^; permutation tests with 1000 permutations were performed to assess model significance^24^.

A meta-taxonomic analysis of the upper respiratory microbiota was performed through oral and nasopharyngeal swabs in asymptomatic (Sars-CoV-2 infected asymptomatic) and symptomatic (Sars-CoV-2 infected symptomatic) pregnant women. First, we evaluated the microbial community structure according to the type of swab.

Then, we characterized the nasopharyngeal microbiota of asymptomatic SARS-CoV-2 infected, symptomatic Sars-CoV-2 infected pregnant women and healthy control subjects by analyzing alpha and beta-diversity (i.e., the bacterial richness and diversity within and between samples, respectively), two parameters used to evaluate the overall microbial ecology.

## Results

### Study population and pregnancy outcomes

A total of 43 pregnant women were enrolled in the study, including 21 non-infected (healthy controls) and 22 SARS-CoV-2-infected participants (13 asymptomatic and 9 symptomatic; Table 1). Only in 37 of the 43 participants was it possible to perform both nasopharyngeal and oral microbiota analysis (18 non-infected, 11 asymptomatic, and 8 symptomatic SARS-CoV-2-infected women). Additionally, in a further 5 patients only the nasopharyngeal (N) swab specimens could be analysed with a total of 42 patients analysed (21 from healthy controls, 12 asymptomatic and 9 symptomatic SARS-CoV-2-infected women), and in only one other patient was it possible to analyse the oral (O) swab specimen, with a total of 38 (18 from healthy controls, 12 asymptomatic and 8 symptomatic SARS-CoV-2-infected women) (Figure S1).

**Table 1:** Demographic and clinical variables of the study population subdivided in non-infected (healthy), asymptomatic (SCoV-2_A) and symptomatic (SCoV-2_S) SARS-CoV-2 infected pregnant women in nasopharyngeal (N) and oral (O) swab.

*N*	Overall population		Healthy		SCoV-2_A		SCoV-2_S	
	*N*	O	*N*	O	*N*	O	*N*	O
	42	38	21	18	12	12	8	8
Age (years)^b^	33 (30.5–37)	33 (30.8–37)	33 (30.5–38)	33 (31-37.5)	32.5 (28.5–36.8)	32 (26.5–36.8)	33 (31-34.8)	33.5 (31.5–34.8)
Ethnicity	42	38	21	18	12	12	9	8
Africa & Middle East	10 (24%)	9 (24%)	2 (10%)	2 (11%)	3 (25%)	3 (25%)	5 (56%)	4 (50%)
Caucasian	27 (64%)	25 (66%)	18 (85%)	16 (89%)	6 (50%)	6 (50%)	3 (33%)	3 (38%)
Asian	2 (5%)	2 (5%)	0	0	2 (17%)	2 (17%)	0	0
Latin American	3 (7%)	2 (5%)	1 (5%)	0	1 (8%)	1 (8%)	1 (11%)	1 (12%)
BMI (Kg/m^2^**)**	26.9 (24.5–29.2)	27 (24.6–29.5)	25.6 (24.3–29.5)	24.9 (24.2–28.9)	27 (24-28.9)	27.6 (26.1–29.3)	27.5 (26.2–30.2)	27.8 (26.3–30.2)
Weight gain (Kg) ^f^	10 (9–14)	10 (7–13)	11 (8-14.5)	10 (7-13.5)	12.5 (10-14.8)	11.5 (10-14.5)	9.5 (7.6–10)	8.5 (7–10)
N. of smokers	2 (5%)	3 (8%)	1 (5%)	1(6%)	1 (8%)	1 (8%)	0	1 (12%)
Gestational age (weeks)^a, c,e, f^	38 (37.5–39)	38 (36.5–39)	38 (38–39)	38 (38–39)	38 (38–40)	38 (37–39)	34 (31–38)	34 (31–38)
Pregnancy complications								
Obesity (BMI > = 30)	8 (20%)	8 (21%)	5 (24%)	4 (22%)	1 (8%)	2 (17%)	2 (25%)	2 (25%)
Diabetes	5 (12%)	5 (13%)	3 (14%)	3 (17%)	1 (8%)	1 (8%)	1 (13%)	1 (13%)
Hypertension	2 (5%)	2 (5%)	2 (10%)	2 (11%)	0	0	0	0
Hypothyroidism	7 (17%)	7 (18%)	3 (14%)	3 (17%)	2 (17%)	2 (17%)	2 (25%)	2 (25%)
Cholestasis	2 (5%)	2 (5%)	0	0	0	0	2 (25%)	2 (25%)
Others	4 (10%)	4 (11%)	1 (5%)	1 (6%)	2 (17%)	3 (25%)	1 (13%)	0
Symptoms								
Gastrointestinal	1 (2%)	1 (3%)	1 (5%)	1 (6%)	0	0	0	0
Mild respiratory	3 (7%)	4 (11%)	0	0	0	0	3 (38%)	4 (50%)
Severe respiratory	4 (10%)	4 (11%)	0	0	0	0	4 (50%)	4 (50%)
Others	1 (2%)						1 (13%)	
N. of patients with with caries in the last 2 yrs	19 (46%)	16 (42%)	11 (52%)	9 (50%)	6 (50%)	5 (42%)	0	2 (25%)
Blood sample								
HB (g/dL)	10.4 (9.1–12.1)	10.3 (9.1–12.1)	9.9 (9.0–11.0)	10.0 (9.0-11.9)	11.4 (9.3–12.1)	10.6 (9.3–11.8)	10.9 (9.8–12.2)	10.9 (9.9–11.7)
WBC (10e9/L) ^a,b^	11 (9.9–12.2)	10.9 (9.9–12.2)	11 (10.4–11.6)	11.0 (10.4–11.4)	12.0 (10.8–14.7)	12.0 (10.8–14.7)	9.1 (7.1–10.9)	8.9 (6.4–9.7)
PCR (mg/dL)^c^	0.9 (0.3–4.2)	0.9 (0.3–4.2)	0.3 (0.3–0.4)	0.3 (0.3–0.4)	1.5 (0.4–3.5)	1.5 (0.4–3.5)	0.9 (0.1-9.0)	0.9 (0.2-6.0)
PCT (ng/mL)	0.07 (0.06–0.12)	0.07 (0.05–0.14)	0.1 (0.1–0.1)	0.1 (0.1–0.1)	0.1 (0.1–0.1)	0.1 (0.1–0.1)	0.1 (0.1–0.2)	0.1 (0.1–0.2)

Data are presented as median (IQR) or n(%) as appropriate.

Oral ^a^: *p* < 0.05 healthy vs. SCoV-2_S; ^b^: *p* < 0.05 SCoV-2_A vs. SCoV-2_S; ^c^: *p* < 0.05 healthy vs. SCoV-2_A.

Nasopharyngeal ^d^: *p* < 0.05 healthy vs. SCoV-2_A; ^e^: *p* < 0.05 healthy vs. SCoV-2_S; ^f^: *p* < 0.05 SCoV-2_A vs. SCoV-2_S.

BMI, body mass index; HB, haemoglobin; WBC, White blood cell; PCR, C-reactive protein; PCT, procalcitonin; IQR, interquartile range.

The characteristics of the study population, along with laboratory test results and pregnancy complications, are detailed in Table 1. The prevalence of pregnancy complications was comparable between infected and non-infected groups. Among the 22 infected women, 9 were symptomatic; of these, 4 (44%) experienced severe symptoms (dyspnea), while 5 (56%) had mild to moderate symptoms (cough, fever). None required admission to the intensive care unit. Notably, the control group exhibited a significantly higher white blood cell count compared to the symptomatic patients.

Perinatal outcomes, summarized in Table 2, did not significantly differ between the groups.

**Table 2:** Demographic and clinical variables of newborns of non-infected (healthy), asymptomatic (SCoV-2_A) and symptomatic (SCoV-2_S) SARS-CoV-2 infected pregnant women. Data are presented as median (interquartile range) or n (%) as appropriate.

	Overall population		Healthy		SCoV-2_A		SCoV-2_S	
	*N*	O	*N*	O	*N*	O	*N*	O
Fetal weight (g)	3140 (2945–3450)	3123 (2095–4270)	3020 (2815–3265)	2999 (2560–3610)	3295 (3146–3485)	3188 (2400–3660)	3268 (2646–4225)	3323 (2095–4270)
pH A	7.26 (7.22–7.29)	7.25(7.13–7.40)	7.26 (7.28–7.37)	7.24 (7.13–7.31)	7.24 (7.23–7.33)	7.23 (7.14–7.27)	7.34 (7.28–7.40)	7.34 (7.28–7.40)
pH V	7.35 (7.28–7.37)	7.33 (7.20–7.40)	7.35 (7.28–7.37)	7.33 (7.2–7.4)	7.36 (7.32–7.38)	7.33 (7.22–7.40)	7.29 (7.25–7.34)	7.29 (7.23–7.38)
BE A	− 1.35 (− 3.18 to − 0.80)	− 2.34 (− 8.1 to 0.7)	-1.60 (− 3.85 to − 0.08)	− 2.38 (− 8.1 to 0.7)	-1.70 (-3.05 - -0.15)	-2.69 (-4.8 -0.2)	-0.9 (-1.0 - -0.8)	-0.9 (-1- -0.8)
BE V	− 1.55 (− 2.08 to − 0.80)	− 1.72 (− 8 to 1.3)	− 1.6 (− 2.3 to − 0.30)	− 1.55 (− 8 to 1.3)	-1.6 (-2.3 - -0.7)	-2.05 (-3.8- -0.3)	-1.15 (-2.43 - -0.80)	-1.57 (-3.7- -0.5)

*A*, artery; *V*, vein; *BE*, base excess; *IQR*, interquartile range.

### Nasopharyngeal and oral microbiota in the overall study population

The meta-taxonomic analysis of the nasopharyngeal and oral microbiota in asymptomatic and symptomatic SARS-CoV-2 positive and negative pregnant women revealed variations in the microbial community structure consistent with the type of swab, reflecting the biogeography of the human respiratory microbiota^25–27^.

**Fig. 1:**
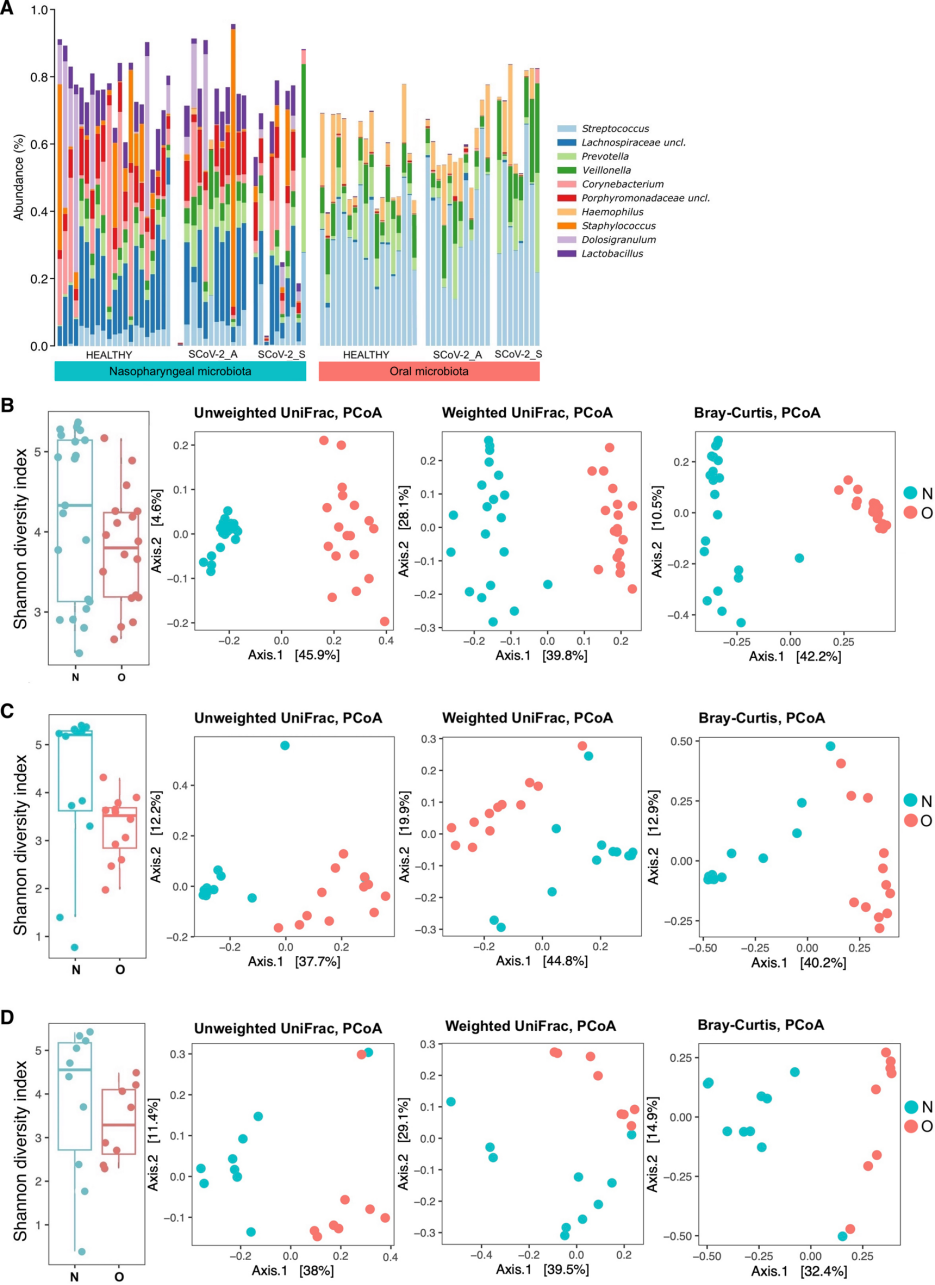
(**A**) Stacked bar plots showing the relative abundance of the 10 most prevalent genera in the nasopharyngeal and oral microbiota of non-infected (healthy), asymptomatic (SCoV-2_A), and symptomatic (SCoV-2_S) SARS-CoV-2 infected pregnant women. Each bar represents an individual sample, with genera ordered by their relative abundance across all samples. (**B**–**D**) Alpha- and beta-diversity analyses measured using the Shannon index, and unweighted, weighted and Bray-Curtis distance matrices in nasopharyngeal (N) vs. oral (O) swabs in (**B**) non-infected (healthy) pregnant women, (**C**) asymptomatic (SCoV-2_A) SARS-CoV-2 infected pregnant women and (**D**) symptomatic (SCoV-2_S) SARS-CoV-2 infected pregnant women. Alpha-diversity was evaluated using the Shannon index. Comparisons of alpha-diversity between nasopharyngeal and oral microbiota were performed using the Wilcoxon rank-sum test (Healthy, *P* = 0.11; SCoV-2_A, *P* = 0.091; SCoV-2_S, *P* = 0.29). Beta-diversity analysis between nasopharyngeal and oral microbiota was conducted using PERMANOVA, for the Unweighted UniFrac distance (Healthy, *P* = 0.001; SCoV-2_A, *P* = 0.001; SCoV-2_S, *P* = 0.003), the Weighted UniFrac distance (Healthy, *P* = 0.001; SCoV-2_A, *P* = 0.001; SCoV-2_S, *P* = 0.001), and the Bray-Curtis dissimilarity index (Healthy, *P* = 0.001, SCoV-2_A, *P* = 0.001; SCoV-2_S, *P* = 0.001).

In the studied population, bacterial variability in the nasopharyngeal microbiota was greater than in the oral microbiota. The most abundant genera in the nasopharyngeal microbiota were: Lachnospiraceae, Corynebacterium, Porphyromonadaceae, Dolosigranulum, Lactobacillus, Staphylococcus, Prevotella, Veillonella, and Streptococcus; while in the oral microbiota, the most abundant genera were primarily Streptococcus followed by Prevotella, Veillonella, and Haemophilus (Fig. 1A and Table S4).

Alpha and beta-diversity analyses demonstrated differences in microbial community structure between nasopharyngeal and oral microbiota (Fig. 1B–D), although no significant differences in alpha-diversity were observed. Comparisons of alpha-diversity between nasopharyngeal and oral microbiota were performed using the Wilcoxon rank-sum test (Healthy, *P* = 0.11; SCoV-2_A, *P* = 0.091; SCoV-2_S, *P* = 0.29).

Beta-diversity analysis between nasopharyngeal and oral microbiota was performed using PERMANOVA for the Unweighted UniFrac distance (Healthy, *P* = 0.001; SCoV-2_A, *P* = 0.001; SCoV-2_S, *P* = 0.003), for the Weighted UniFrac distance (Healthy, *P* = 0.001; SCoV-2_A, *P* = 0.001; SCoV-2_S, *P* = 0.001) and the Bray-Curtis dissimilarity index (Healthy, *P* = 0.001, SCoV-2_A, *P* = 0.001; SCoV-2_S, *P* = 0.001).

### Changes in the nasopharyngeal microbiota in SARS-CoV-2 infected pregnant women

**Fig. 2:**
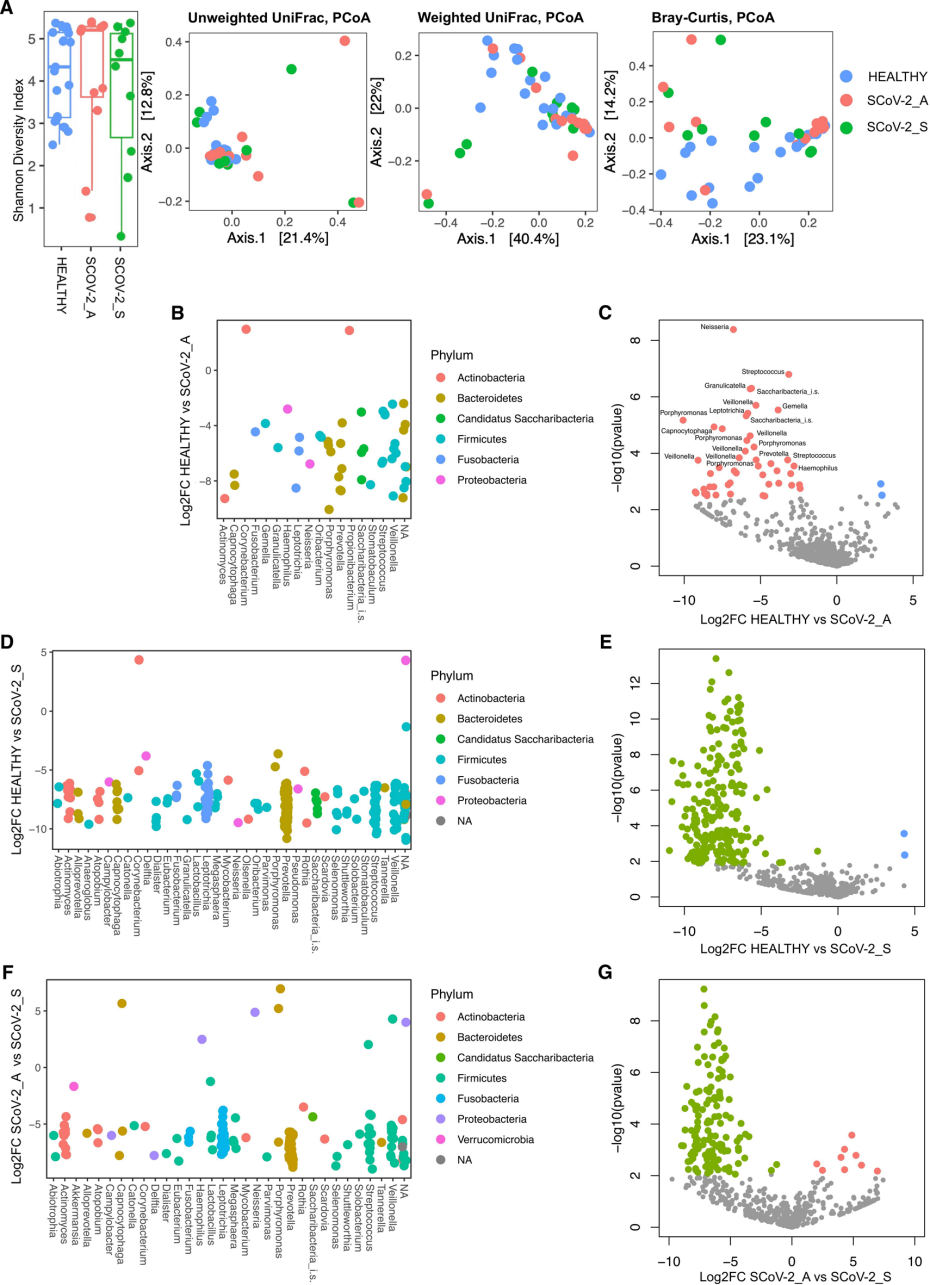
(**A**) Alpha- and beta-diversity analysis of the nasopharyngeal microbiota in non-infected (healthy), asymptomatic (SCoV-2_A), and symptomatic (SCoV-2_S) SARS-CoV-2 infected pregnant women, measured using the Shannon index (Wilcoxon rank-sum test, *P* = 0.64 for all three categories), Unweighted UniFrac distance (PERMANOVA, *P* = 0.056), Weighted UniFrac distance (PERMANOVA, *P* = 0.259), and Bray-Curtis dissimilarity index (PERMANOVA, *P* = 0.056) matrices. (**B**, **D**,**F**) Genus vs. log2FC plots of significant ASVs; (**C**, **E**, **G**) Volcano plots showing significantly enriched bacterial ASVs (FDR *p* < 0.05) in comparisons of (**B**, **C**) healthy vs. SCoV-2_asymptomatic, (**D**, **E**) healthy vs. SCoV-2_symptomatic, and (**F**, **G**) SCoV-2_asymptomatic vs. SCoV-2_symptomatic, based on the DEseq2 analysis of the nasopharyngeal microbiota. The names of significantly enriched ASVs classified to the genus level with FDR *p* < 0.01 are reported only for healthy vs. SCoV-2_asymptomatic.

The nasopharyngeal microbiota of SARS-CoV-2 infected pregnant women (both asymptomatic and symptomatic) and healthy control women was studied. The alpha and beta-diversity analyses showed no significant differences among the groups, although the results were close to statistical significance (alpha diversity: Wilcoxon rank-sum test, *p* > 0.05; beta diversity: PERMANOVA, *p* = 0.056 for the Unweighted UniFrac distance and *p* = 0.056 for the Bray-Curtis dissimilarity index) (Fig. 2A).

In the nasopharyngeal microbiota, the analysis of microbial community structure according to clinical data shows that the alpha-diversity (i.e. the within sample diversity) showed no significant differences according to the different clinical variables taken into account (i.e. ethnicity, BMI, pathologies, past surgeries, drug usage, allergies, smoke, dental caries last 2 years, comorbidities) (pairwise Wilcoxon rank-sum test, *p* > 0.05).

The analysis of beta-diversity (i.e. the between samples diversity) showed a significant difference in the microbial community structure according to smoking and allergies by using the Unweighted UniFrac distance (PERMANOVA, *p* < 0.05) and according to smoking and ethnicity by using the Bray-Curtis dissimilarity index (PERMANOVA *p* < 0.05); therefore, ethnicity and allergies contributed to shaping the microbiota regardless of the infection status of the host.

Overall, these data suggest that the nasopharyngeal microbial community structure is not significantly affected by the health status of the patients, showing only minor differences close to statistical significance.

Further analysis revealed variations in microbial taxa between the nasopharyngeal microbiota of asymptomatic, symptomatic, and healthy pregnant women. Both asymptomatic and symptomatic women showed an enrichment of pathogens and pathobionts such as Corynebacterium, Fusobacterium, Neisseria, Streptococcus, Haemophilus, Mycobacterium and Porphyromonas compared to the control group.

Asymptomatic pregnant women showed an enrichment of pathobionts such as Neisseria, Streptococcus, Haemophilus and Porphyromonas compared to the control group (Fig. 2B,C, and Table S5).

Symptomatic patients were enriched in pathobionts such as Campylobacter, Fusobacterium, Mycobacterium, Neisseria and Porphyromonas compared to the control group (Fig. 2D,E, and Table S6). Compared to asymptomatic women, symptomatic patients showed enrichment in pathobionts such as Campylobacter, Corynebacterium, Fusobacterium, Haemophilus, Mycobacterium, Neisseria and Porphyromonas, Streptococcus (Fig. 2F,G, and Table S7).

### Changes in the oral microbiota in SARS-CoV-2 infected pregnant women

**Fig. 3:**
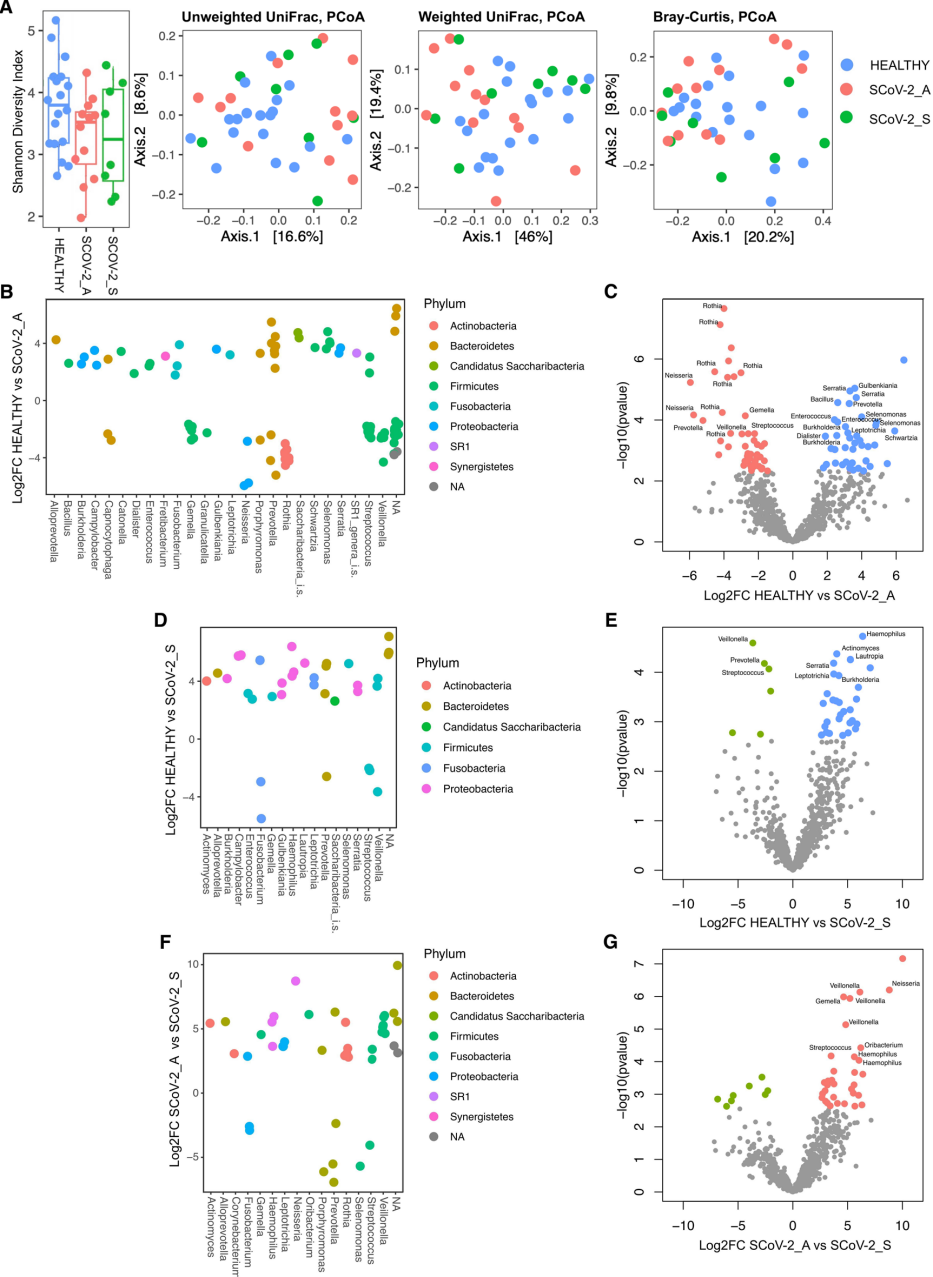
(**A**) Alpha- and beta-diversity analysis of non-infected (healthy), asymptomatic (SCoV-2_A), and symptomatic (SCoV-2_S) SARS-CoV-2 infected oral microbiota as measured using the Shannon index (Wilcoxon rank-sum test, *P* = 0.31 Healthy vs. SCoV-2_A, and Healthy vs. SCoV-2_A; *P* = 0.94 SCoV-2_A vs. SCoV-2_S), Unweighted UniFrac distance (PERMANOVA, *P* = 0.043), Weighted UniFrac distance (PERMANOVA, *P* = 0.174), and Bray-Curtis distance (PERMANOVA, *P* = 0.312) matrices in oral swabs. **B**, **D**, **F**) Genus vs. log2FC plot of the significant ASVs, and **C**, **E**, **G**) Volcano plots representing the significantly enriched bacterial ASVs (FDR *p* < 0.05) in (**B**, **C**) healthy vs. SCoV-2_asymptomatic, (**D**, **E**) healthy vs. SCoV-2_symptomatic and **F**, **G**) SCoV-2_asymptomatic vs. SCoV-2_symptomatic by the DEseq2 analysis of the oral microbiota. The names of the significantly enriched ASVs classified at the genus level with FDR *p* < 0.01 are also reported.

The alpha-diversity analysis showed no significant differences among groups using different ecological indices (pairwise Wilcoxon rank-sum test, *p* > 0.05). The analysis of beta-diversity based on patients’ health status showed a significant difference in microbial community structure using the Unweighted UniFrac distance (PERMANOVA, *p* = 0.043), while no differences were observed using the Weighted UniFrac and Bray-Curtis metrics (PERMANOVA *p* > 0.05) (Fig. 3A).

In the oral microbiota, the analysis of alpha-diversity (i.e. the within sample diversity) showed no significant differences according to the different clinical variables taken into account (i.e. ethnicity, BMI, pathologies, past surgeries, drug usage, allergies, smoke, dental caries last 2 years, comorbidities) (pairwise Wilcoxon rank-sum test, *p* > 0.05).

Additionally, significant differences in microbial community structure were observed according to the ethnic origin of patients using the Weighted UniFrac distance (PERMANOVA, *p* < 0.05).

A variation in microbial taxa was observed between the oral microbiota of the three study groups (asymptomatic and symptomatic SARS-CoV-2 infected pregnant women and healthy group): both asymptomatic and symptomatic pregnant women showed an enrichment of pathobionts such as Neisseria, Fusobacterium, and Streptococcus. Asymptomatic women showed enrichment of pathobionts such as Neisseria and Streptococcus compared to the control group (Fig. 3B,C, and Table S8). Symptomatic patients showed enrichment of pathobionts such as Fusobacterium and Streptococcus compared to the control group (Fig. 3D-E, and Table S9).

Regarding the differences between asymptomatic and symptomatic women, many pathobionts such as Streptococcus, Fusobacterium and Porphyromonas were enriched in both groups, while other potential pathogens such as Haemophilus and Neisseria were more abundant only in asymptomatic women (Fig. 3F,G, and Table S10).

### Best predictor of SARS-CoV-2 infection between nasopharyngeal and oral microbiota in pregnant women

**Fig. 4:**
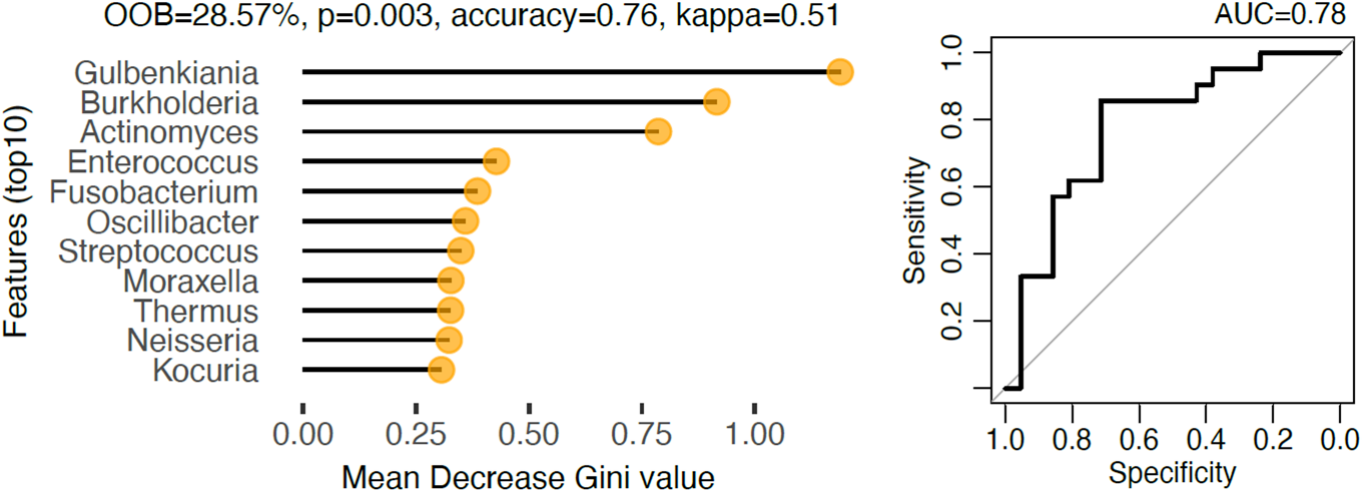
The top 10 most important bacterial genera for classifying SARS-CoV-2 positive versus negative pregnant women in nasopharyngeal swabs and their respective ROC curve.

To better understand which sampling method would better predict the infection status of the patients, we used a random-forest classifier on metagenomics data from nasopharyngeal and oral swabs. With the AUROC of 0.78 and an out-of-bag (OOB) error rate of 28.6%, the classifier showed a significant predictive power (permutation tests *p*-value = 0.003, accuracy = 76%, kappa = 52%) for the nasopharyngeal microbiota (Fig. 4).

The most important features selected by the classifier were *Gulbenkiania*, *Burkholderia*, and *Actinomyces*, among others, all taxa were significantly enriched in control group compared to SARS-CoV2 infected pregnant women. In contrast, the oral microbiota was a poorer predictor of patients infection status (OOB = 41%, permutation tests p-value = 0.117, accuracy = 57%, kappa = 15%).

However, when trained to classify patients as symptomatic COVID, asymptomatic COVID, or healthy controls, the classifier performed poorly in predicting infection severity showing an OOB error rate of 52.63% (*p* = 0.083) for the oral site and 55.81% (*p* = 0.05) for the nasopharyngeal site.

The 10 most important genera for classifying SARS-CoV-2 infection vs. controls in nasopharyngeal microbiota are: *Gulbenkiania*,* Burkholderia*,* Actinomyces*,* Enterococcus*,* Fusobacterium*,* Oscillibate*,* Streptococcus*,* Moraxella*,* Thermus*,* Neisseria*,* and Kocuria*.

### Correlation of the nasopharyngeal and oral microbiota with pregnant women’s clinical parameters

In addition, we correlated the relative abundance of the most prevalent genera (with a mean relative abundance > 0.1%) identified in the nasopharyngeal and oral microbiota with the different clinical parameters of pregnant women. *Dolosigranulum* and *unclassified Neisseriaceae* genera correlated positively with increased body weight in pregnancy.

**Fig. 5:**
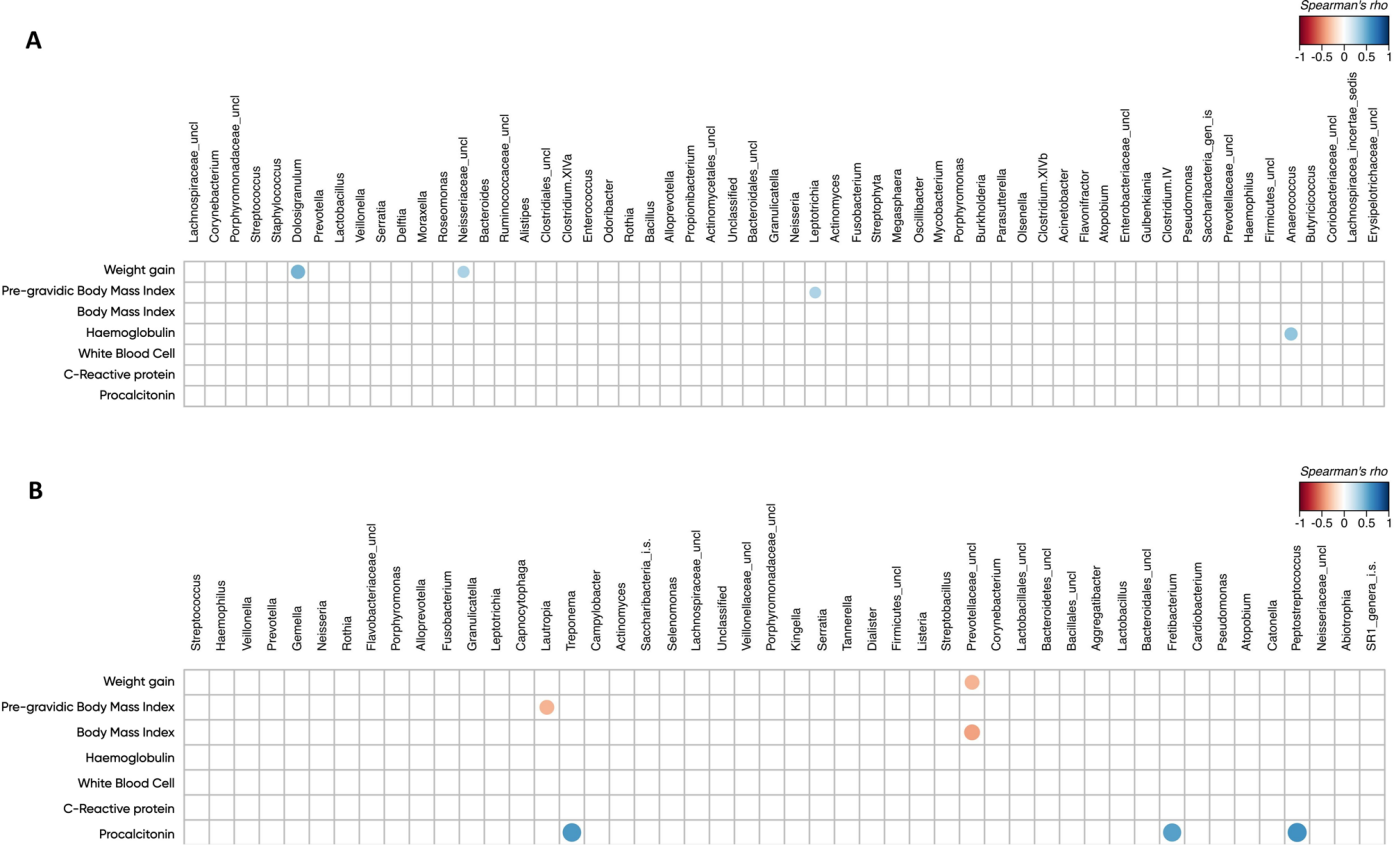
Correlation between clinical data and the most abundant bacterial genera (mean relative abundance > 0.1%) for (**A**) nasopharyngeal microbiota and (**B**) oral microbiota. Solid circles represent the degree of correlation among the variables considered, with an FDR-corrected *p* < 0.05.

On the other hand, we found a negative correlation of *unclassified Prevotellaceae* with BMI and body weight gain. Lastly, *Treponema*,* Peptosteptococcus* and an unclassified genera of *Bacteroidales* positively correlated with blood procalcitonin (PCT) levels (*p* < 0.05). Altogether these data suggest that the nasopharyngeal and oral microbiota of SARS-CoV-2 infected pregnant women correlates with their inflammatory and gestational weight gain (Fig. 5A,B).

## Discussion

In the present study we investigated the changes of nasopharyngeal and oral microbiota in SARS-CoV-2 infected and non-infected pregnant women. To our knowledge, this is the first study to have simultaneously assessed changes in the nasopharyngeal and oral microbiota during SARS-CoV-2 infection, during the first waves of this syndemic, prior to the availability of SARS-CoV-2 vaccines.

In agreement with current knowledge, in our study population, the analysis of the alpha and beta-diversity, two parameters used to evaluate the overall microbial ecology, confirmed the different structure of microbial community associated with the nasopharyngeal and oral swabs.

The analysis of alpha and beta-diversity of the nasopharyngeal microbiota showed only minor differences, but no significant differences in SARS-CoV-2 infected pregnant patients and healthy pregnant women. To the best of our knowledge, our findings of the microbiota of the upper respiratory in pregnant patients were not markedly different from the general population according to the presence or absence of SARS-CoV2 infection.

Overall, these data suggest that the microbial community structure of nasopharyngeal microbiota is not widely affected by the health status of the patients. However, a microbial biodiversity reduction was found in the SARS-CoV-2 pregnant group with a relative increase of pathobiont genera acquisition according to the severity of symptomatology.

Pregnant nasopharyngeal communities were dominated by Firmicutes phylum, followed by *Bacteroidetes*, *Proteobacteia* and *Actinobacteria*. Crovetto et al.^28^ finds the same dominant communities except for *Bacteroidetes*. At the genus level, *Steptocococcus* was the most abundant genera followed by *Corynebacterium* and *Staphylococcus*. Crovetto found the last two genera to be the most abundant. Similar to Crovetto, we found an enrichment at the level of the *Bacteroidetes* phylum and of the *Prevotellaceae* and *Lachnospiraceae* families in SarsCoV-2 infected pregnant women compared to controls.

Unlike Crovetto we found significant differences when SARS-CoV-2 infected pregnant women were stratified into symptomatic and asymptomatic. In fact, symptomatic cases were enriched by specific pathobionts: *Campylobacter*, *Fusobacterium* and *Mycobacterium* while the latter have the following ones in common: *Neisseria* and *Porphyromonas* suggesting that SARS-CoV-2 disease progression and severity affects the composition of the respiratory microbiota and its relationship with possible respiratory tract coinfections.

Similarly to nasopharyngeal microbiota, oral microbiota showed small differences in the overall structure of microbial community as measured by alpha and beta-diversity among symptomatic and asymptomatic SARS-CoV-2 infected and non-infected pregnant women. However, similarly to nasopharyngeal microbiota, both asymptomatic and symptomatic pregnant women were enriched by pathobionts such as *Neisseria*, *Fusobacterium* and *Streptococcus* when compared to healthy group, but not any difference was observed between the symptomatic-asymptomatic patients. In addition to this, the nasopharyngeal microbiota correlated with ethnic group and previous health conditions like smoking attitude and allergies, while the oral one correlated limitedly with ethnic group.

There were no alpha diversity differences between SARS-CoV-2 infected patients and healthy groups, however the oral microbiota composition showed differences between the two groups, as already reported in the previous research by Leftwich et al.^29^. The peculiar oral cavity stability could be associated with the wash-out phenomena typical of that anatomical district.

By simultaneously analyzing the oral and nasopharyngeal microbiota, it was possible to conclude that the nasopharyngeal microbiota seems to be a better predictor of SARS-CoV-2 infection and its severity than oral one.

These data suggests that SARS-CoV2 infection favours the colonisation of pathobionts affecting the commensal resident population of the nasopharyngeal cavity. The oral microbiota was a poorer predictor of SARS-CoV2 infection status.

We found additional correlations between the relative abundance of the most abundant genera identified in the nasopharyngeal and oral microbiota with several clinical parameters of pregnant women.

Altogether, these data suggest that the nasopharyngeal and oral microbiota of SARS-CoV2 infected pregnant women correlates with their inflammatory status and gestational weight gain. In addition, the observed increase of procalcitonin values - related to the genera *Treponema*, *Peptosteptococcus* and unclassified *Bacteroidales*—could be provoked by the related endothelial damage caused by COVID-19 syndrome.

In our cohort we did not observe any specific correlation between the SARS-CoV-2 infection and pregnancy complications or newborn status. In our study, we found no differences between the groups analysed with regard to perinatal outcomes, which is probably due to the fact that the women, although symptomatic, did not have a serious infection and because an average of four days elapsed from the positive swab with hospitalisation to delivery.

The strength of this work was mainly due to the methodology of the oral and nasopharyngeal swabs taken at the time of hospital admission, in order to exclude changes in the microbiota due to hospitalisation. We also excluded women who had taken antibiotics in the previous month in order to avoid confounding factors. Similarly, the duration of recruitment was set to exclude the possible influence of mass vaccination in pregnancy. The gestational age window from 31 to 40 weeks allowed studying only the changes in the microbiota due possibly to SARS-CoV-2 infection and not those caused by gestational age differences. The number of cases recruited was relatively small, but we obtained for each pregnant woman all the obstetrical data, and the clinical data to classify the severity of the SARS-CoV-2 infection. The relatively small sample size did not allow for additional stratifications according to ethnicity and socio-economic status.

Similarly to previous clinical studies, we only collected samples in infected patients and pre-existing alterations in their microbiota could not be excluded.

To confirm the above mentioned hypothesis, in order to expand our biotic network epistemology, whereas each individual homeostasis is far beyond a single agent presence according to the ancient and Gestaltic principle: “the whole is more than the sum of the individual parts”.

## Conclusion

SARS-CoV-2 infected pregnant women revealed an alteration in the nasopharyngeal and oral microbiota compared to healthy pregnant ones.

We found a variation in taxa, represented by a pathobionts enrichment in both nasopharyngeal and oral microbiota of the SARS-CoV-2 infected pregnant women, significantly increased in symptomatic cases.

The nasopharyngeal microbiota appears to be a better predictor of SARS-CoV-2 infection and its severity than the oral one.

Based on these results, future studies may confirm whether the nasopharyngeal microbiota is also a better predictor of infection for other respiratory viral infections than the oral microbiota and whether the healthy microbiota has a role as a modulator of the immune response to viral infections in pregnancy.

## Supplementary Information

Below is the link to the electronic supplementary material.

Supplementary Material 1

Supplementary Material 2

Supplementary Material 3

Supplementary Material 4

Supplementary Material 5

Supplementary Material 6

Supplementary Material 7

Supplementary Material 8

Supplementary Material 9

Supplementary Material 10

Supplementary Material 11

Supplementary Material 12

## Data Availability

Sequencing raw data, and de-identifier clinical data are deposited in the Zenodo data-base 10.5281/zenodo.11173791. The data that support the findings of this study are available from the corresponding author, [Lattuada D.], upon reasonable request.
