# Supplementary material for: Nasopharyngeal and oral microbiota profiling in SARS-CoV-2 infected pregnant women
Source: Sci Rep. 2025 Oct 9;15:35306. doi: 10.1038/s41598-025-19344-5 (PMC12511543; doi:10.1038/s41598-025-19344-5)
Supplement: Supplementary file 11 — Supplementary Material 11 [file 41598_2025_19344_MOESM11_ESM.docx]

**Figure S1 : Study population**


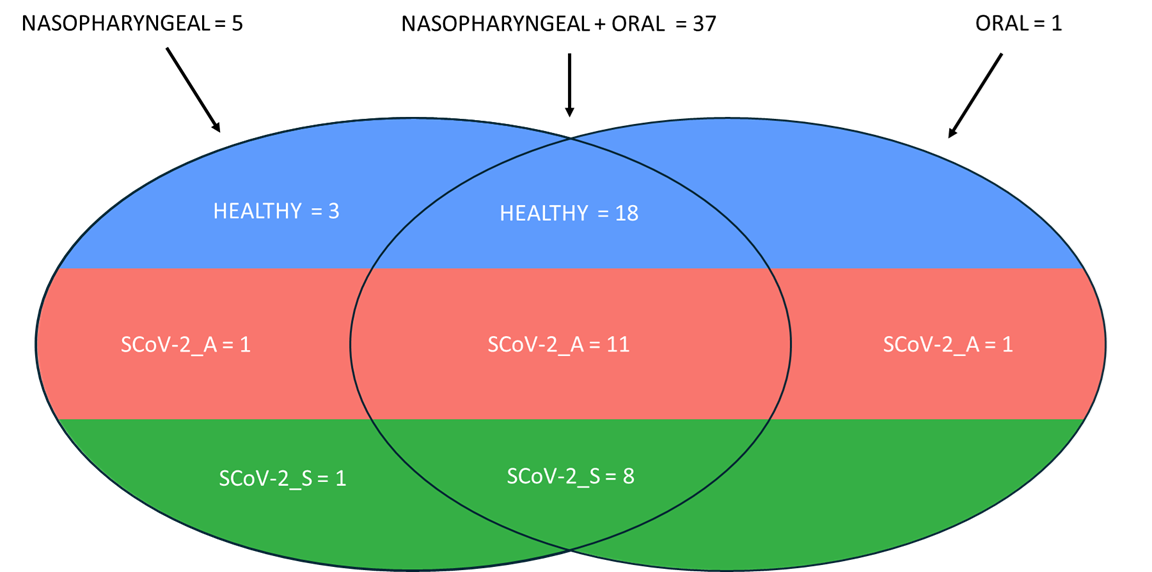


A total of 43 pregnant women were enrolled in the study, including 21 non-infected (healthy controls ●) and 22 SARS-CoV-2-infected participants (13 asymptomatic ● and 9 symptomatic ●).

Only in 37 of the 43 participants was it possible to perform both nasopharyngeal and oral microbiota analysis (18 non-infected, 11 asymptomatic, and 8 symptomatic SARS-CoV-2-infected women). Additionally, in a further 5 patients only the nasopharyngeal (N) swab specimens could be analysed with a total of 42 patients analysed (21 from healthy controls, 12 asymptomatic and 9 symptomatic SARS-CoV-2-infected women), and in only one other patient was it possible to analyse the oral (O) swab specimen, with a total of 38 (18 from healthy controls, 12 asymptomatic and 8 symptomatic SARS-CoV-2-infected women).
